# Tracking the Time-Dependent Role of the Hippocampus in Memory Recall Using DREADDs

**DOI:** 10.1371/journal.pone.0154374

**Published:** 2016-05-04

**Authors:** Carmen Varela, Sarah Weiss, Retsina Meyer, Michael Halassa, Joseph Biedenkapp, Matthew A. Wilson, Ki Ann Goosens, Daniel Bendor

**Affiliations:** 1 Picower Institute for Learning and Memory, Department of Brain and Cognitive Sciences, MIT, Cambridge, MA, United States of America; 2 McGovern Institute for Brain Research, Department of Brain and Cognitive Sciences, MIT, Cambridge, MA, United States of America; 3 The Neuroscience Institute, Langone Medical Center, New York University, New York, New York, United States of America; 4 Institute of Behavioral Neuroscience, Department of Experimental Psychology, University College London, London, United Kingdom; Nathan Kline Institute and New York University Langone Medical Center, UNITED STATES

## Abstract

The hippocampus is critical for the storage of new autobiographical experiences as memories. Following an initial encoding stage in the hippocampus, memories undergo a process of systems-level consolidation, which leads to greater stability through time and an increased reliance on neocortical areas for retrieval. The extent to which the retrieval of these consolidated memories still requires the hippocampus is unclear, as both spared and severely degraded remote memory recall have been reported following post-training hippocampal lesions. One difficulty in definitively addressing the role of the hippocampus in remote memory retrieval is the precision with which the entire volume of the hippocampal region can be inactivated. To address this issue, we used Designer Receptors Exclusively Activated by Designer Drugs (DREADDs), a chemical-genetic tool capable of highly specific neuronal manipulation over large volumes of brain tissue. We find that remote (>7 weeks after acquisition), but not recent (1–2 days after acquisition) contextual fear memories can be recalled after injection of the DREADD agonist (CNO) in animals expressing the inhibitory DREADD in the entire hippocampus. Our data demonstrate a time-dependent role of the hippocampus in memory retrieval, supporting the standard model of systems consolidation.

## Introduction

Although hippocampal lesions impair the ability to recall recent episodic experiences, more remote memories are typically spared [1–4]. While this would suggest that the hippocampus is not required to retrieve a remote memory, several cases of hippocampal lesions in both humans and rodents have led to a disruption of remote memories [5–7]. One potential caveat to lesion-based data is the proportion and precision of the hippocampal damage. If only part of the hippocampus is lesioned, the ability to recall remote memories might arise because the remaining, unaffected portion of the hippocampus is functionally sufficient for memory recall. On the other hand, lesions that lead to deficits in remote memory recall may be extremely large, extending from the hippocampus to neighboring neocortical regions. Because neocortical regions are thought to be important for the storage of remote memories [8], the deficits in remote memory retrieval could be due to the inactivation of regions outside of the hippocampus, confounding the interpretation of the observed memory deficits. While neuroanatomical targeting can be used to create large, relatively accurate lesions, new molecular genetic tools (e.g. optogenetics and chemogenetics) provide two additional advantages- 1) reversibility, and 2) precise targeting of neurons based on a genetic-profile (e.g. cell-type, cortical layer, or brain region) [9–11]. Chemogenetics imparts an additional advantage for studying hippocampal function over time, the capacity to reversibly manipulate a large brain volume without the need for any implants, which would be required with optogenetics. Activation of the chemogenetic receptor is accomplished through the delivery of a ligand capable of reaching the entire brain via the circulatory system, with the manipulation limited to neurons expressing the chemogenetic receptor. Instead, previous hippocampal inactivation studies using molecular genetic tools such as optogenetics have been limited to the dorsal hippocampus, due to the technical difficulty of illuminating the entire hippocampus with optical fibres. [12]. Here we use DREADDs (Designer Receptors Exclusively Activated by Designer Drugs) [13] to chemogenetically disrupt activity, both remotely and reversibly, throughout both the dorsal and ventral hippocampus of mice during the recall of a contextual fear memory. We find that DREADDs-based hippocampal manipulation leads to a blockade of recent (1–2 day old) but not remote (>7 weeks old) memories, adding to the evidence [12,14–16] in support of the time-dependent role of the hippocampus in memory retrieval.

## Materials and Methods

We used C57BL/6J mice (30–40 gr) in this study (Jackson Laboratory; Bar Harbor, ME); mice were housed individually, received food and water ad libitum, and were monitored by veterinarian staff in a temperature-controlled room with a 12 h light/dark cycle (lights on/off at 7:00 am/7:00 pm). These experiments were approved by the Committee on Animal Care at the Massachusetts Institute of Technology, and conform to US National Institutes of Health guidelines for the care and use of laboratory animals.

### DREADDs vector and viral injections

**Fig 1:**
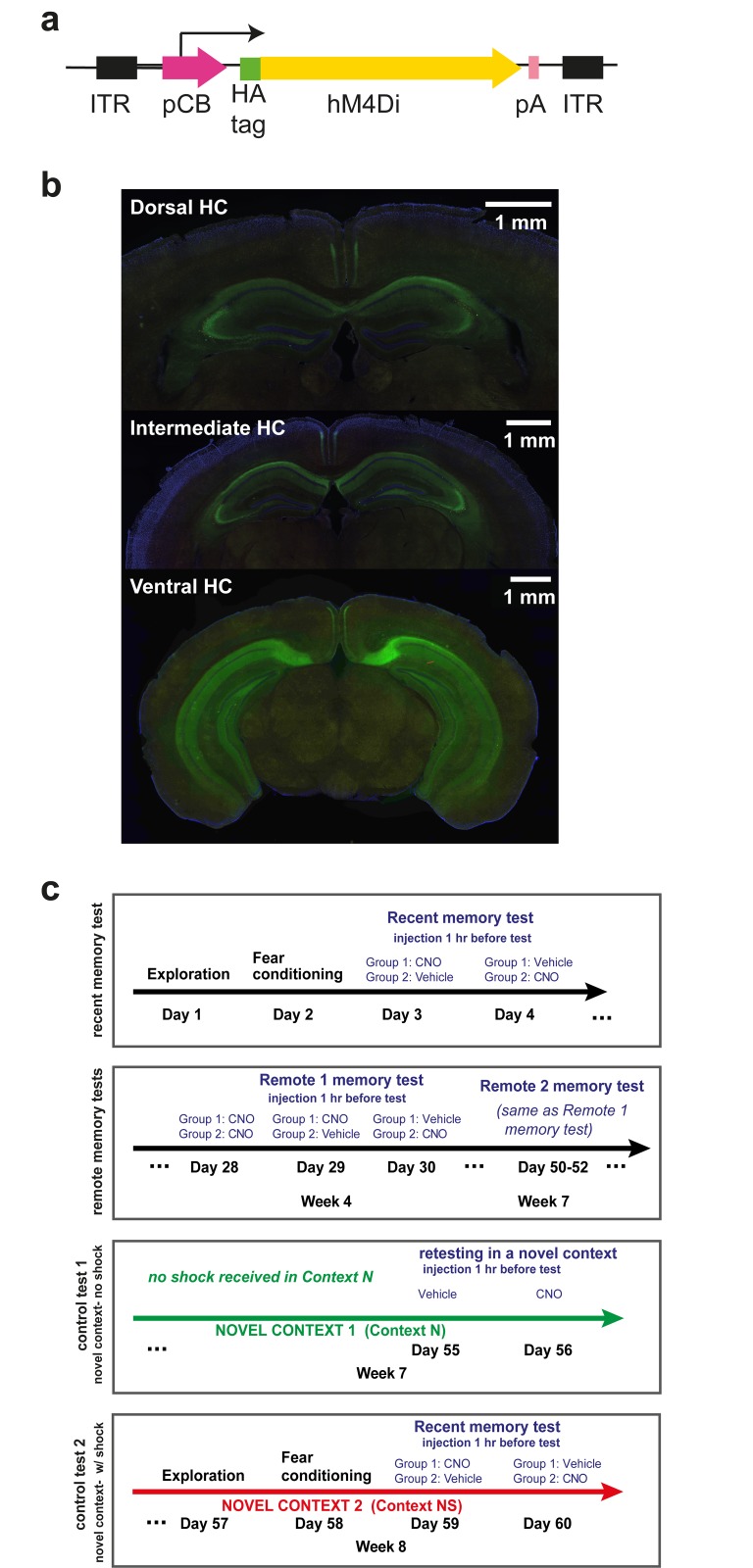
Experimental methods and histological confirmation of DREADD expression. a: AAV construct for hM_4_Di expression in vivo. The open reading frame of hM_4_Di contains a Hemagglutinin (HA)-tag at its 5’ end and a poly-A (PA) tail on its 3’ end. This gene is driven by an ubiquitous pCB promoter. ITR = inverted terminal repeats. Expression of the HA tag thus directly reflects levels of the hM_4_Di protein expression. b: hM_4_Di expression (green) in dorsal, intermediate, and ventral hippocampus in a representative mouse brain (A3) revealed by immunohistochemistry (see text for details). Image was acquired using an AxioCam HR camera on an Axio Imager Z1 motorized microscope (Zeiss) with a standard Zeiss FITC filter set. c: Fear conditioning protocol: From top row to bottom row- Recent memory test in Context 1(top row), Remote 1 and 2 memory test in Context 1(second row), test in Context N (third row), and Recent memory test in Context NS (bottom row).

The DREADDs variant used in our experiments was a modified inhibitory human muscarinic G-protein coupled receptor (hM_4_Di), engineered to respond to the drug Clozapine-N-Oxide (CNO) without responding to its native ligand, acetylcholine. CNO has two desirable properties as a ligand: 1) it is inert within the brain, it binds selectively to DREADD receptors and is not metabolized to Clozapine in the rodent at low concentrations, eliminating also secondary interactions with other receptors, and 2) it crosses the blood-brain barrier, permitting dosing orally or via intraperitoneal injections [17]. We expressed the hM_4_Di receptor (directly fused to hemagglutinin—HA- for histological confirmation) in the hippocampus of wild-type mice (C57BL/6) using an AAV-9 vector (>1 x 10^11^ infectious particles/mL), under the control of a non-specific pCB promoter (Fig 1a). The vector was injected during sterile stereotaxic surgery; briefly, animals were anesthetized using 2% isoflurane in oxygen, followed by maintenance with 1% inhaled isoflurane. Using four stereotaxically targeted injections per animal (coordinates from the Paxinos & Watson atlas: Dorsal hippocampus: -2.2 AP, ±2 L, 1.4 D, 1 μl/infusion; Ventral Hippocampus: -3.2 AP, ±3.5 L, 3 D; 1 μl/infusion), we were able to achieve expression over the full dorsal and ventral extent of the CA1, CA3, and dentate gyrus subregions of the hippocampus (Fig 1b, n = 12). After surgery, animals were left to recover in a clean home cage placed on a heated pad; buprenorphine hydrochloride (Buprenex, 0.015 mg/kg) was provided for analgesia on the day of the surgery and in the following three days post-surgery.

### Contextual fear conditioning

Four weeks after surgery, a time point when receptor expression is maximal and stable [18], mice were trained in a contextual fear conditioning protocol. Twelve age-matched mice with no virus injections served as a control group. Before the initiation of the behavioral experiment, animals were habituated to transport from the animal facility and handling by both experimenters for at least three days prior to the onset of training. A contextual fear conditioning protocol began the next day (for timeline, see Fig 1c). Each day animals were transported from the animal facility to a quiet holding room at least one hour before the experiment started. On Day 1, all mice were placed in the conditioning context for 5 minutes [Context 1: 0.3% Pine-Sol odor, illumination from room and overhead chamber lights, ambient noise provided by a small fan in each chamber] in a sound-attenuated chamber (MED Associates; St. Alban, VT). On Day 2, animals were conditioned in the same context. After 3 minutes of exploration, they received 5 mild shocks (0.7mA, 1s, variable inter-shock intervals of 60-90s).

All subsequent tests consisted of re-exposing the animals to the conditioning context for 90 seconds with no shock delivered. Recent memory tests occurred on Days 3 and 4; the mice were briefly re-exposed (90s total) to the same context to assess freezing behavior, a measure of the retrieval strength of the fear memory; the brevity of the test sessions was designed to minimize memory extinction between the test sessions. The context memory tests were conducted one hour after receiving an intraperitoneal (i.p.) injection of either CNO (dissolved at 1mg/ml in 0.01M PBS; dosage: 5mg/kg) or Vehicle (0.01M PBS). A cross-over design was used to test all mice with CNO and Vehicle injections: half of the mice received CNO on Day 3 and Vehicle on Day 4, and the other half received injections in the reverse order. Context-induced fear was measured by assessing freezing behavior (defined by complete lack of movement save for respiration) offline using commercial software (VideoFreeze, MED Associates). This is a robust measure of the strength of the fear memory [19]. Freezing was measured starting 30 seconds after the mouse was placed in the context until it was removed (60 seconds total). The freezing level threshold was manually set to the mean level determined by two experimenters who were blind to the experimental conditions and identity of the subjects.

This same group of mice were tested again at two additional time points for recall of fear in context 1: *Remote 1* (4 weeks after training) and *Remote 2* (7 weeks after training). These time points were selected because other studies have suggested that contextual memories are hippocampus-independent at these time points [15–16]. Each remote memory test (90s duration) was conducted across three days. On the first day, all mice received a CNO injection one hour before testing (dosage as in the Recent Memory Test). On the second and third days, mice received either a CNO or Vehicle injection (counterbalanced as for the Recent Memory Test), also one hour before testing. Thus over the three days of each test, each mouse was tested twice after a CNO injection and once after a Vehicle injection.

Three days after the conclusion of the second remote memory test, mice were tested in two different novel contexts, which served as control memory tests. First, mice were retested in a novel context that was not associated with a prior shock [Context N: Ammonia scent, no ambient fan noise, no grid floor, illumination provided by ceiling lights, but no side lights, circular Plexiglas inserts]. In these two sessions (Days 55–56), freezing levels were measured during a 90s exposure to Context N, one hour after receiving an IP injection of either Vehicle (first day) or CNO (second day), with dosage as in first context. Exposure to this context allowed us to test for changes in exploration levels (e.g., due to fear generalization) with and without CNO-mediated hippocampus blockade.

Following testing in Context N, the mice were shocked in a second novel context [Context NS: 1% Acetic acid, illumination provided only by a small stimulus light in each chamber, no ambient fan noise, pyramidal black Plexiglas insert]. The recall of the new recent fear memory of context NS was subsequently tested one hour following CNO and Vehicle injections to ascertain the effectiveness of DREADDs inactivation at this time point. Briefly, all mice were pre-exposed to Context NS for 5 minutes (Day 57), and, on the following day (Day 58), returned to context NS for contextual fear conditioning (5 unsignaled footshocks, as per Fig 1c). During the next two days following training, the mice were re-exposed to Context NS (90s) to assess freezing levels. Freezing levels were measured one hour after receiving an IP injection of either CNO or Vehicle (with dosage as in first context). Similar to the previous tests, CNO and Vehicle injection order was counterbalanced, with half of the animals receiving CNO on the first test day and Vehicle on the second test day, and in reverse order for the other half of the mice.

Video files of recent and remote memory tests are archived on Figshare at https://figshare.com/s/e8c9fd732e9d66024293.

### Spontaneous alternation task

A single trial consisted of placing the mouse at the entrance of the central arm of a T-maze, where they could then explore either the left or right arm. Upon returning to the central arm, a blockade was placed in front of the animal to hold him at the base of the central arm, and the barrier was removed after a brief delay (up to 10 seconds). Upon the removal of the blockade, the mouse then visited either the same arm as visited previously or the opposite arm. A visit to the opposite arm was recorded as a spontaneous alternation. Each mouse was tested in 3 CNO and 3 Vehicle sessions across 6 days, with each session consisting of 12 trials.

### Histology

Expression of the hM_4_Di receptor was confirmed post-mortem. After completing behavioral testing, mice were anesthetized with isoflurane and given an overdose of sodium pentobarbital; once the animal stopped breathing, it was transcardially perfused with 0.01 M PBS followed by 4% paraformaldehyde. Brains were post-fixed overnight before obtaining coronal sections (50–60μm) with a vibratome (Leica VT1000S). Brain sections were run through an immunohistochemistry protocol in order to reveal the hemagglutinin (HA) tag fused to the hM_4_Di receptor [Steps: 1) blocking 1h with 5% goat serum + 2.5% Bovine Serum Albumin + 0.1% TritonX in 0.01M PBS; 2) Primary antibody (High affinity anti-HA, from Roche), 1:200, 24h at 4 C; 3) Secondary antibody tagged with green fluorescent protein (Invitrogen product number A11006), 1:1000, 2-3h at room temperature].

## Results

**Fig 2:**
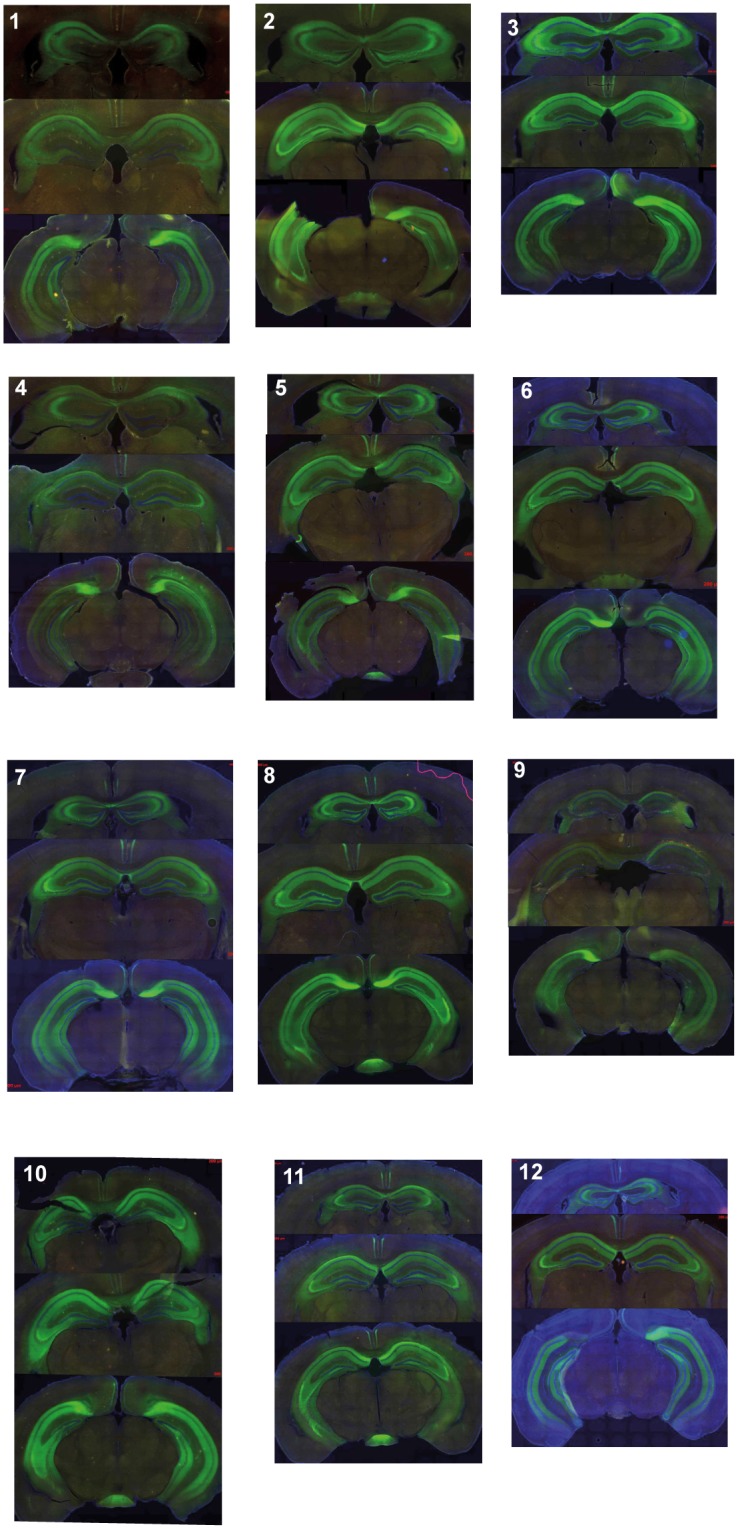
hM_4_Di expression. hM_4_Di expression (green) in dorsal, intermediate, and ventral hippocampus in all fear conditioned hM4Di mice. Mice A1-A12 labelled 1–12. Images were acquired using an AxioCam HR camera on an Axio Imager Z1 motorized microscope (Zeiss) with a standard Zeiss FITC filter set.

**Fig 3:**
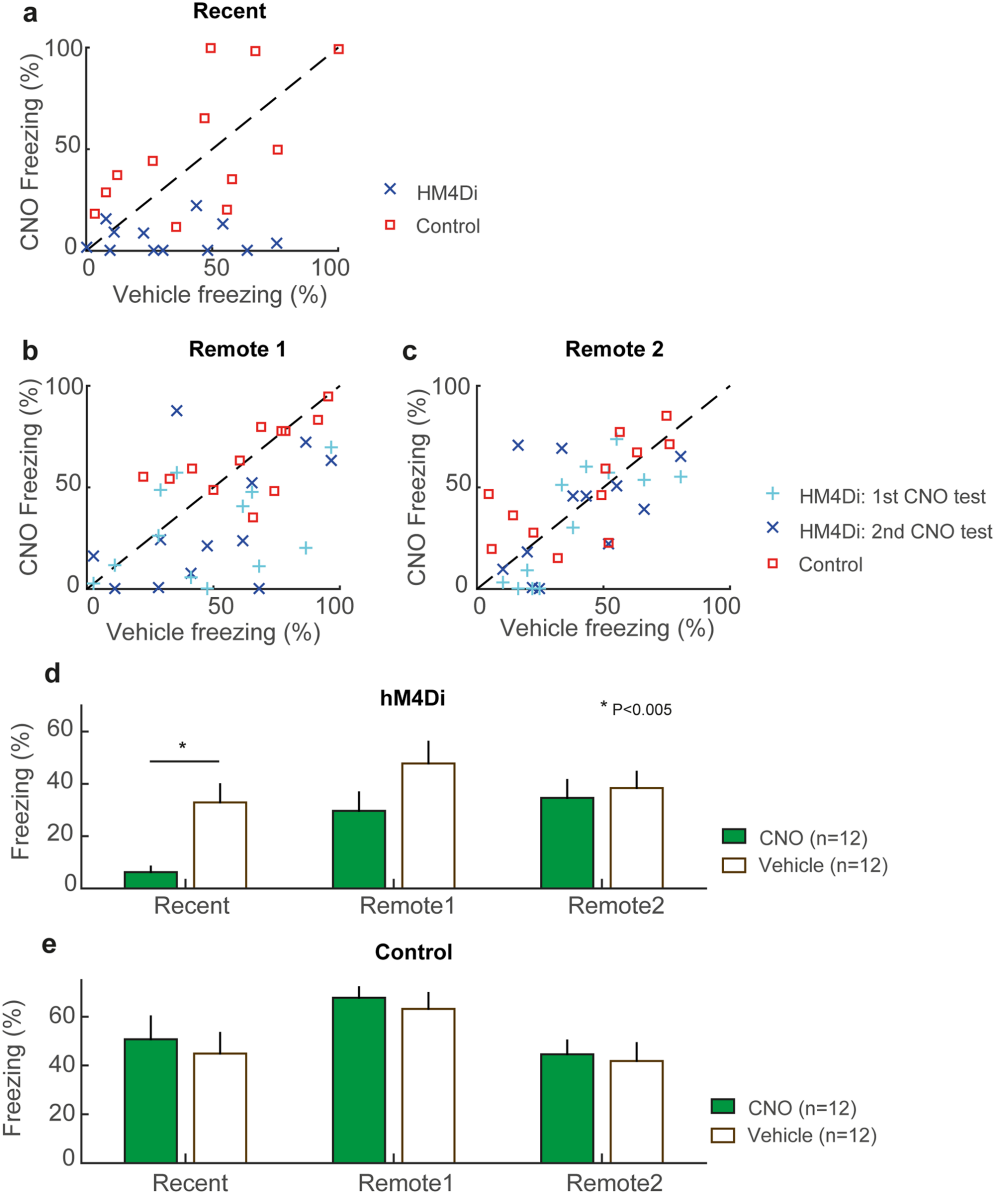
Testing the hippocampus dependence of recent and remote memories. a-c: Comparison of freezing levels (post-CNO and post-Vehicle injection) in the (a) recent memory test, (b) remote 1 memory test, (c) remote 2 memory test, for control mice (n = 12, red) and hM_4_Di mice (n = 12, blue, cyan). d-e: Freezing levels following CNO (filled bars) and Vehicle (unfilled bars) injections for the Recent, Remote 1 and Remote 2 memory test in (d) hM_4_Di mice and (e) control mice. (* P<0.005, Signed rank test). For the Remote 1 and Remote 2 tests, the mean CNO freezing level (across two successive tests) is plotted).

We compared memory retrieval between hM_4_Di-expressing and control mice that had previously undergone contextual fear conditioning (representative example of hM_4_Di expression in Fig 1b, also see Fig 2 and Materials and Methods), using freezing level as a proxy for the recall strength of the contextual fear memory. Mice were initially tested 1–2 days after receiving several shocks in their testing box (Context 1, see Materials and Methods). We found that hM_4_Di-expressing mice had lower freezing levels during re-exposure to the fear-conditioned context after an injection of the hM_4_Di-specific ligand CNO than after Vehicle injections (Fig 3a, 3d and 3e, S1 and S2 Videos). Within-subject freezing levels were, on average, 26.7% lower after an injection of CNO, compared to levels following injection of Vehicle, a decrease that was statistically significant (mean freezing levels- CNO: 6.2%, Vehicle: 32.9%, Fig 3a and 3d, Signed rank test, P<0.005). In contrast, control mice with no hM_4_Di expression had near equivalent mean freezing levels whether administered with Vehicle (44.9%) or CNO (50.7%) (Fig 3a and 3e, Signed rank test, P = 0.73). The disruption of memory recall occurred only in mice expressing hM_4_Di in the hippocampus after receiving CNO, indicating that 1–2 days post training, the contextual fear memory was still hippocampus-dependent.

Using the same group of mice, we tested the strength of the contextual fear memory again at two later time points: *Remote 1* (4 weeks after training) and *Remote 2* (7 weeks after training), each spanning three test days. On the first day of each Remote test, all mice received a CNO injection one hour before exposure to the context. On the second and third days, mice received either a CNO or Vehicle injection one hour before exposure to the context (counterbalanced as with the Recent Memory Test). Thus for both the Remote 1 and Remote 2 time points, mice received two CNO tests, contrasting the single CNO test used during the recent memory time point. The use of two CNO tests allowed all mice to receive CNO before their first exposure to the context at the remote time points (prior to being tested with Vehicle), and to receive a second CNO test counterbalanced with a Vehicle test. This was done to examine whether reinstating the memory (during the Vehicle injection test) prior to the CNO test affected the hippocampus-dependence of the memory.

For the *Remote 1* memory test (Fig 3b and 3d), the mean freezing level in hM_4_Di-expressing mice was 28.5% in the first CNO test and 30.8% in the second CNO test, compared to after a Vehicle injection (47.8%). However, these freezing levels were not statistically significant for either the first CNO test (Signed rank test, P = 0.064, uncorrected) or second CNO test (Signed rank test, P = 0.064, uncorrected). The mean freezing levels of control mice (Fig 3b and 3e) were not significantly different between CNO and Vehicle injections- first CNO test: 70.7%, second CNO test: 64.7%, Vehicle test: 63.2%, (Signed rank test: first CNO test: P = 0.57, uncorrected, second CNO test: P = 0.79, uncorrected).

For the *Remote 2* Memory Test (Fig 3c, 3d and 3e, S3 and S4 Videos), the mean freezing level in hM_4_Di-expressing mice was 32.8% in the first CNO test and 36.5% in the second CNO test, compared to a freezing level of 38.4% after a Vehicle injection. This difference between both CNO tests and the vehicle test was not statistically significant (Signed rank test, first CNO test: P = 0.30, second CNO test: P = 0.57, uncorrected). The mean freezing levels of control mice (Fig 3c and 3e) were also not significantly different between CNO and Vehicle injections- first CNO test: 41.2%, second CNO test: 48.0%, Vehicle test: 41.8% (Signed rank test, first CNO test: P = 0.97, second CNO test: P = 0.23, uncorrected).

While these data suggest that the hippocampus is not required to recall a contextual fear memory at 7 weeks post-acquisition, it is possible that multiple re-exposures to a now safe context influenced the animal’s memory trace, which has been avoided in previous fear conditioning studies by performing only a single test. To reduce this possibility, context exposures at each test were limited to 90 seconds. Furthermore, we observed no evidence of extinction of the contextual fear memory, as freezing levels were stable over Recent, Remote 1, and Remote 2 Vehicle testing sessions in both control and hM_4_Di animals (Kruskal-Wallis test, hM_4_Di: P = 0.37, control: P = 0.11; Fig 3c and 3d).

**Fig 4:**
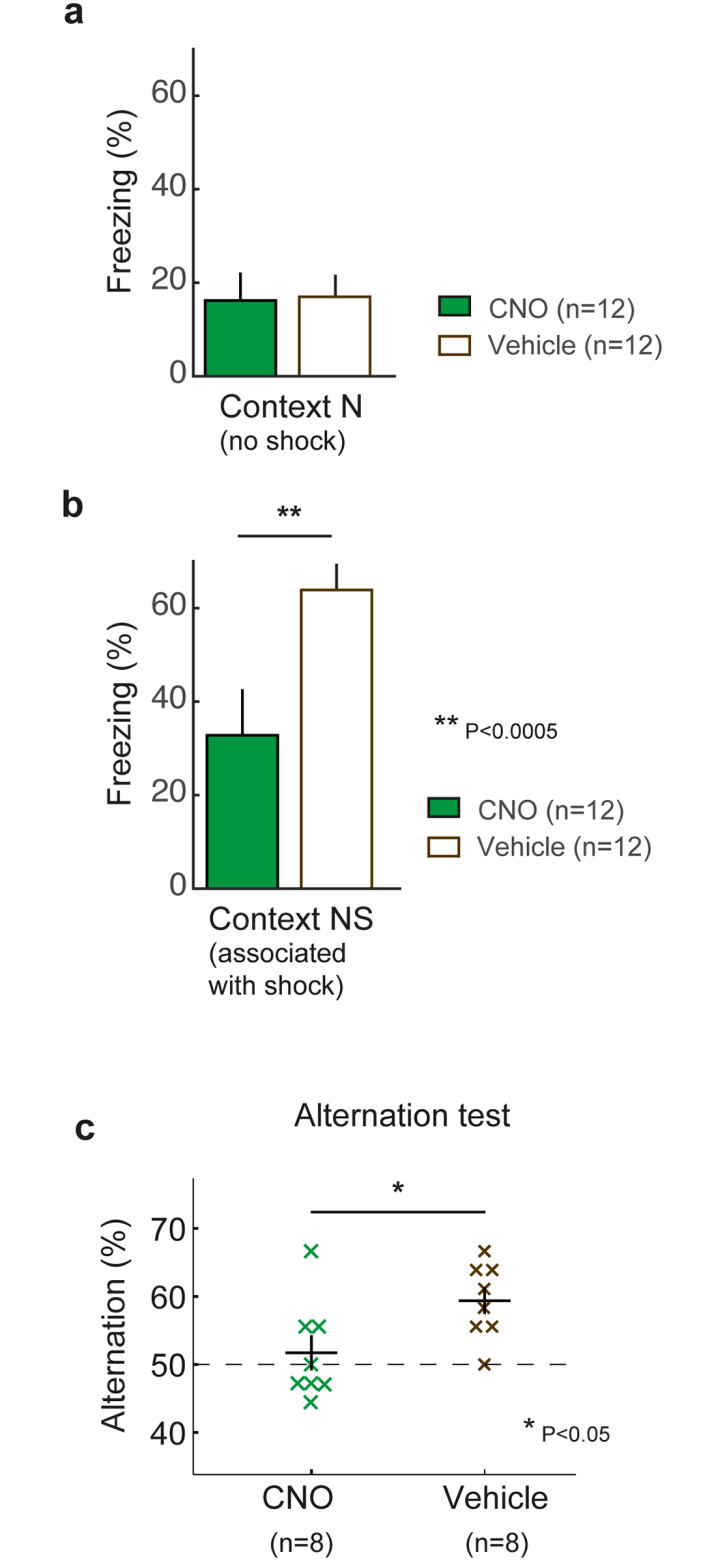
Testing the efficacy of hippocampal inactivation in hM_4_Di mice. a: Freezing levels in hM_4_Di mice following CNO and Vehicle injections in Context N ([P = 1, Signed rank test, n = 12]. No shock had been previously given in Context N. b: Freezing levels in hM_4_Di mice following CNO and Vehicle injections in Context NS (previously paired with a shock) [*P<0.0005, Signed rank test, n = 12]. c: Percent alternation in a T-maze spontaneous alternation task in hM_4_Di mice (n = 8) after CNO (green) and Vehicle (brown) injections.

Another possibility is that the context-specificity of the fear memory has changed over time, and the lack of hippocampus dependence at remote time points reflects a generalized expression of fear when the context specific information stored in the hippocampus is not available (i.e., during CNO). We next examined whether the remote fear memory was specific for Context 1, or had generalized and could be observed in a novel context that lacked a fear association. Three days after the conclusion of the second remote memory test, the same group of mice were retested in a novel context that was not associated with a prior shock [Context N, see Materials and Methods]. During these two days (Days 55–56), freezing levels were measured during a 90s exposure to Context N, one hour after receiving an IP injection of either Vehicle (first day) or CNO (second day). Mean freezing levels were similar in both the CNO (16.2%) and Vehicle (17.0%) test (Fig 4a, Signed rank test, P = 1). Although these were non-zero freezing levels, indicating that the memory had undergone some degree of generalization, freezing levels were significantly less than what was experienced in Context 1 during the Remote2 memory test (mean freezing levels across CNO and Vehicle conditions, Remote2 = 37.4%, Context N = 16.6%, Signed rank test, P<0.003). Because freezing levels in Context N test were similar with the CNO and Vehicle tests (Signed rank test, P = 1), this suggests that the specificity of the remote memory did not depend on an intact hippocampus.

Another alternative interpretation of our data is that expression of the hM_4_Di receptor decreased with the passage of time, and CNO was no longer able to suppress the recall of a hippocampus-dependent memory. To verify that CNO was still actionable and capable of inducing a behavioral effect, the same mice underwent new fear learning in a second novel context [Context NS, see Materials and Methods], after completing the context N tests (Day 57). During the Context NS tests, hM_4_Di-expressing mice had lower freezing levels after an injection of the hM_4_Di-specific ligand CNO compared to after Vehicle injections, a difference that was statistically significant (CNO: 32.8%, Vehicle: 63.9%, Signed rank test, P<5.0x10^-4^, Fig 4b). These data indicate that CNO still effectively disrupted the recall of a recent (1–2 day old) contextual fear memory in hM_4_Di-expressing mice three months after viral transduction (covering the entire duration of our fear conditioning protocol using context 1 and context N).

Our experiments using contextual fear conditioning indicate that the disruption of hippocampal activity produces temporally graded amnesia, with the recall of more remote memories (>7 weeks) no longer requiring a functional hippocampus. One potential issue in interpreting these data is that while changes in freezing levels correlate with the strength of a fear memory, this behavior could be modified by other factors. For example, hyperlocomotion can result from large hippocampal lesions [20], however, we did not observe that low freezing levels were the byproduct of hyperlocomotion (S1, S2, S3 and S4 Videos).

Although we successfully targeted the hippocampus in all of our hM4Di mice, two mice (subjects 4 and 9) showed substantially weaker expression (Fig 2). We repeated our analysis excluding subjects 4 and 9, and observed similar results across recent, remote2, and novel context tests (S1 Table). The one exception to this was observed for the first remote test, where we observed a statistically significant difference in freezing levels following a CNO injection, compared with a Vehicle injection (first test P<0.05, second test, P<0.02, Signed rank test, uncorrected).

As an additional control to test whether the DREADDs manipulation directly affected hippocampal function, we tested an additional group of 8 hM_4_Di mice in a spontaneous alternation task, a hippocampus-dependent behavior that relies on working memory but not motor activity levels [21]. One hour prior to testing, hM_4_Di-expressing mice were given either a CNO or Vehicle injection as in the fear conditioning experiments. We observed a mean spontaneous alternation rate of 52% after a CNO injection compared to a spontaneous alternation rate of 59% alternation following a Vehicle injection (Fig 4c). hM_4_Di-expressing mice had a significantly lower rate of spontaneous alternation (Signed rank test, P<0.05), and effectively chose between arms of the behavioural apparatus at chance levels following a CNO injection (compared to a Vehicle injection), suggesting disrupted hippocampal function.

## Discussion

In this study, we have utilized a chemogenetic technique via expression of inhibitory DREADDs to reversibly test the time-dependent role of the hippocampus on contextual fear memory retrieval. We observed that the injection of CNO blocked the retrieval of a recent memory (1–2 days post-acquisition), but not a remote memory (7 weeks post-acquisition) in animals that expressed the hM_4_Di receptor in the hippocampus. At intermediate time points (4 weeks post-acquisition), we observed a disruption in memory retrieval in a subset of mice (Fig 3b). However, while mean freezing levels were qualitatively decreased by hippocampus inactivation, this effect was not statistical significant at this time point (P>0.05). However, when we excluded two hM4Di mice with the weakest expression levels from this analysis (S1 Table), we did observe a weak, but statistically significant (P<0.05) CNO-dependent decrease in mean freezing levels during the Remote 1 test (4 weeks post-acquisition). It is possible that multiple re-exposures to the now safe context influenced the animal’s memory trace, which has been avoided in previous fear conditioning studies by limiting subjects to a single test. However, we did not observe any evidence of extinction resulting from multiple re-exposures to the same context, as freezing levels were stable over Recent, Remote 1, and Remote 2 Vehicle testing sessions in both control and hM_4_Di animals (Fig 3d and 3e). Additionally, the fact that CNO had different effects on contextual fear at different time points suggests that the impairment in fear memory observed at earlier time points cannot be attributed to non-specific effects of the drug. Such effects would be observed at all time points.

Following these experiments, the ability to still suppress hippocampal function using CNO was demonstrated by using CNO to successfully block the retrieval of a new recent memory (Context NS) in the same group of mice. Using DREADDs, we have demonstrated a time-dependent role of the hippocampus in memory retrieval, where recent but not remote memories are hippocampus-dependent. It is interesting to consider what brain structures might mediate remote memory retrieval. One possibility is that uninfected hippocampal cells (not expressing hM_4_Di) may have enabled hippocampal participation in remote memory retrieval. While we cannot completely rule this out, remote memory retrieval is associated with a decrease in immediate early gene expression in the hippocampus [15], and both pharmacological and AAV-mediated optogenetic silencing of the hippocampus lead to significantly decreased immediate early gene expression [12,15]. Therefore, a more likely candidate for the retrieval of remote memory is the neocortex, which is selectively recruited to participate in remote memories that were initially hippocampus [12,14–16].

An alternative interpretation of our data is that the hippocampus stores the context-specific details of the remote fear memory [22]. Without an intact hippocampus, the remaining consolidated cortical memory trace is postulated to be a generalized fear memory, lacking a specific contextual association. To address this viewpoint, we measured the freezing level of the hM4Di mice in Context N, a novel context for the mice, which lacked a fear association (the mice were never shocked in this context). Freezing levels in Context N test were not significantly different between CNO and Vehicle tests (Fig 4a, Signed rank test, P = 1), indicating that the specificity of the remote memory was similar both with and without an intact hippocampus. Thus, our data do not support a memory consolidation model where the hippocampus stores the context-specific details of the remote fear memory [22]. We did observe non-zero freezing levels in the Context N test, indicating that the remote fear memory had undergone some degree of generalization. However, our data suggest that the remote memory still retained some degree of specificity, as freezing levels were significantly less in Context N than compared to the Remote2 memory test in Context 1 (Figs 3d and 4a, Signed rank test, P<0.003).

It is important to note that our experiments do not utilize the full capabilities of a chemogenetic approach to targeted inactivation, namely genetically targeting the hippocampus, as our experiments were performed in wild-type mice using a vector with a non-specific promoter, and only used stereotaxically targeted surgery to restrict the expression to the hippocampus. To completely rule out any non-hippocampal DREADDs expression would require using a transgenic mouse model [23], providing specificity for hippocampal neurons while preventing accidental targeting of neighbouring neocortical brain regions. However, a key strength of our approach, lacking from previous studies, is that by using DREADDs we have been able to both track the hippocampal contribution over time within the same subjects, in the same set of hippocampal neurons, and to confirm using histology that the entire dorsal-ventral axis of the hippocampus was affected by the manipulation. Our results help demonstrate the time-dependent role of the hippocampus in memory retrieval, further supporting the standard model of systems memory consolidation [1].

## Supporting Information

S1 Table P-Values for Signed Rank test between CNO and Vehicle freezing levels. A group comparison with and without the exclusion of subjects 4 and 9 (weakest expression levels).
(PDF)

S1 Video Recent memory test after CNO injection. Subject 5, day 3.
(MP4)

S2 Video Recent memory test after Vehicle injection. Subject 5, day 4.
(MP4)

S3 Video Remote2 memory test after CNO injection. Subject 5, day 51.
(MP4)

S4 Video Remote2 memory test after Vehicle injection. Subject 5, day 52.
(MP4)

## References

[1] Squire LR, Alvarez P (1995) "Retrograde amnesia and memory consolidation: A neurobiological perspective". Current Opinion in Neurobiology 5 (2): 169–177. 7620304 10.1016/0959-4388(95)80023-9

[2] Scoville WB, Milner B (1957) Loss of recent memory after bilateral hippocampal lesions. Journal of neurology, neurosurgery, and psychiatry, 20(1), 11 13406589 10.1136/jnnp.20.1.11 PMC497229

[3] Bayley PJ, Hopkins RO, Squire LR (2003) Successful recollection of remote autobiographical memories by amnesic patients with medial temporal lobe lesions. Neuron, 38(1), 135–144. 12691671 10.1016/s0896-6273(03)00156-9

[4] Anagnostaras SG, Maren S, Fanselow MS (1999) Temporally graded retrograde amnesia of contextual fear after hippocampal damage in rats: within-subjects examination. The Journal of neuroscience, 19(3), 1106–1114. 9920672 10.1523/JNEUROSCI.19-03-01106.1999 PMC6782148

[5] Nadel L, Moscovitch M (1997) Memory consolidation, retrograde amnesia and the hippocampal complex. Current Opinion in Neurobiology 7 (2): 217–227. 9142752 10.1016/s0959-4388(97)80010-4

[6] Rempel-Clower NL, Zola SM, Squire LR, Amaral DG (1996) Three cases of enduring memory impairment after bilateral damage limited to the hippocampal formation. The Journal of Neuroscience, 16(16), 5233–5255. 8756452 10.1523/JNEUROSCI.16-16-05233.1996 PMC6579309

[7] Lehmann H, Lacanilao S, Sutherland RJ (2007) Complete or partial hippocampal damage produces equivalent retrograde amnesia for remote contextual fear memories. European Journal of Neuroscience, 25(5), 1278–1286. 17355254 10.1111/j.1460-9568.2007.05374.x

[8] Frankland PW, Bontempi B (2005) "The organization of recent and remote memories". Nature Reviews Neuroscience 6 (2): 119–130. 15685217 10.1038/nrn1607

[9] Rogan SC, Roth BL (2011) Remote control of neuronal signaling. Pharmacological reviews, 63(2), 291–315. 10.1124/pr.110.003020 21415127 PMC3082452

[10] Spiers HJ, Bendor D (2014) Enhance, delete, incept: Manipulating hippocampus-dependent memories. Brain research bulletin, 105, 2–7. 10.1016/j.brainresbull.2013.12.011 24397964 PMC4058530

[11] Fenno L, Yizhar O, Deisseroth K (2011) The development and application of optogenetics. Annual review of neuroscience, 34, 389–412. 10.1146/annurev-neuro-061010-113817 21692661 PMC6699620

[12] Goshen I, Brodsky M, Prakash R, Wallace J, Gradinaru V, Ramakrishnan C, Deisseroth K (2011) Dynamics of retrieval strategies for remote memories. Cell, 147(3), 678–689. 10.1016/j.cell.2011.09.033 22019004

[13] Zhu H, Pleil KE, Urban DJ, Moy SS, Kash TL, Roth BL (2014) Chemogenetic inactivation of ventral hippocampal glutamatergic neurons disrupts consolidation of contextual fear memory. Neuropsychopharmacology, 39(8). 1880–1892. 10.1038/npp.2014.35 24525710 PMC4059896

[14] Lesburguères E, Gobbo OL, Alaux-Cantin S, Hambucken A, Trifilieff P, Bontempi B (2011) Early tagging of cortical networks is required for the formation of enduring associative memory. Science, 331(6019), 924–928. 10.1126/science.1196164 21330548

[15] Maviel T, Durkin TP, Menzaghi F, Bontempi B (2004) Sites of neocortical reorganization critical for remote spatial memory. Science 305:96–99. 15232109 10.1126/science.1098180

[16] Squire LR, Clark RE, Knowlton BJ (2001) Retrograde amnesia. Hippocampus 11, 50–55. 11261772 10.1002/1098-1063(2001)11:1<50::AID-HIPO1019>3.0.CO;2-G

[17] Dong S, Rogan SC, Roth BL (2010) Directed molecular evolution of DREADDs: a generic approach to creating next-generation RASSLs. Nature protocols, 5(3), 561–573. 10.1038/nprot.2009.239 20203671

[18] Aschauer DF, Kreuz S, Rumpel S (2013) Analysis of transduction efficiency, tropism and axonal transport of AAV serotypes 1, 2, 5, 6, 8 and 9 in the mouse brain. PLoS One. 8(9):e76310 10.1371/journal.pone.0076310 24086725 PMC3785459

[19] Blanchard DC, Blanchard RJ (1972) Innate and conditioned reactions to threat in rats with amygdaloid lesions. Journal of comparative and physiological psychology, 81(2), 281 5084445 10.1037/h0033521

[20] Bannerman DM, Yee BK, Good MA, Heupel MJ, Iversen SD, Rawlins JNP (1999) Double dissociation of function within the hippocampus: a comparison of dorsal, ventral, and complete hippocampal cytotoxic lesions. Behavioral neuroscience, 113(6), 1170 10636297 10.1037//0735-7044.113.6.1170

[21] Lalonde R (2002) The neurobiological basis of spontaneous alternation. Neuroscience & Biobehavioral Reviews, 26(1), 91–104. 11835987 10.1016/s0149-7634(01)00041-0

[22] Winocur G, Moscovitch M, Sekeres M (2007) Memory consolidation or transformation: context manipulation and hippocampal representations of memory. Nature neuroscience, 10(5), 555–557. 17396121 10.1038/nn1880

[23] Nakashiba T, Cushman JD, Pelkey KA, Renaudineau S, Buhl DL, McHugh TJ, et al (2012) Young dentate granule cells mediate pattern separation, whereas old granule cells facilitate pattern completion. Cell, 149(1), 188–201. 10.1016/j.cell.2012.01.046 22365813 PMC3319279

