# Supplementary material for: Tracking the Time-Dependent Role of the Hippocampus in Memory Recall Using DREADDs
Source: PLoS One. 2016 May 4;11(5):e0154374. doi: 10.1371/journal.pone.0154374 (PMC4856306; doi:10.1371/journal.pone.0154374)
Supplement: S1 Table — A group comparison with and without the exclusion of subjects 4 and 9 (weakest expression levels). (PDF) [file pone.0154374.s001.pdf]

Supplementary Table

P-Values for Signed Rank test between CNO and Vehicle freezing levels

	**All hM4Di mice (n=12)**	**All hM4Di mice except subjects 4 and 9 (n=10)**
**Recent: CNO vs Vehicle**	**0.0049**	**0.0059**
**Remote 1: CNO (first test) vs Vehicle**	0.064	**0.0488**
**Remote 1: CNO (second test) vs Vehicle**	0.064	**0.0137**
**Remote 2: CNO (first test) vs Vehicle**	0.3013	0.1934
**Remote 2: CNO (second test) vs Vehicle**	0.5693	0.375
**N context: CNO vs Vehicle**	1	0.9453
**NS context: CNO vs Vehicle**	**0.000488**	**0.002**

note: bold values are statistically significant (P<0.05). All P-values are uncorrected.
